# Predicting how surface texture and shape combine in the human visual system to direct attention

**DOI:** 10.1038/s41598-021-85605-8

**Published:** 2021-03-17

**Authors:** Zoe Jing Xu, Alejandro Lleras, Simona Buetti

**Affiliations:** University of Illinois, 603 E. Daniel St., Champaign, IL 61820 USA

**Keywords:** Psychology, Human behaviour, Visual system, Object vision

## Abstract

Objects differ from one another along a multitude of visual features. The more distinct an object is from other objects in its surroundings, the easier it is to find it. However, it is still unknown how this distinctiveness advantage emerges in human vision. Here, we studied how visual distinctiveness signals along two feature dimensions—shape and surface texture—combine to determine the overall distinctiveness of an object in the scene. Distinctiveness scores between a target object and distractors were measured separately for shape and texture using a search task. These scores were then used to predict search times when a target differed from distractors along both shape and texture. Model comparison showed that the overall object distinctiveness was best predicted when shape and texture combined using a Euclidian metric, confirming the brain is computing independent distinctiveness scores for shape and texture and combining them to direct attention.

## Introduction

**Figure 1:**
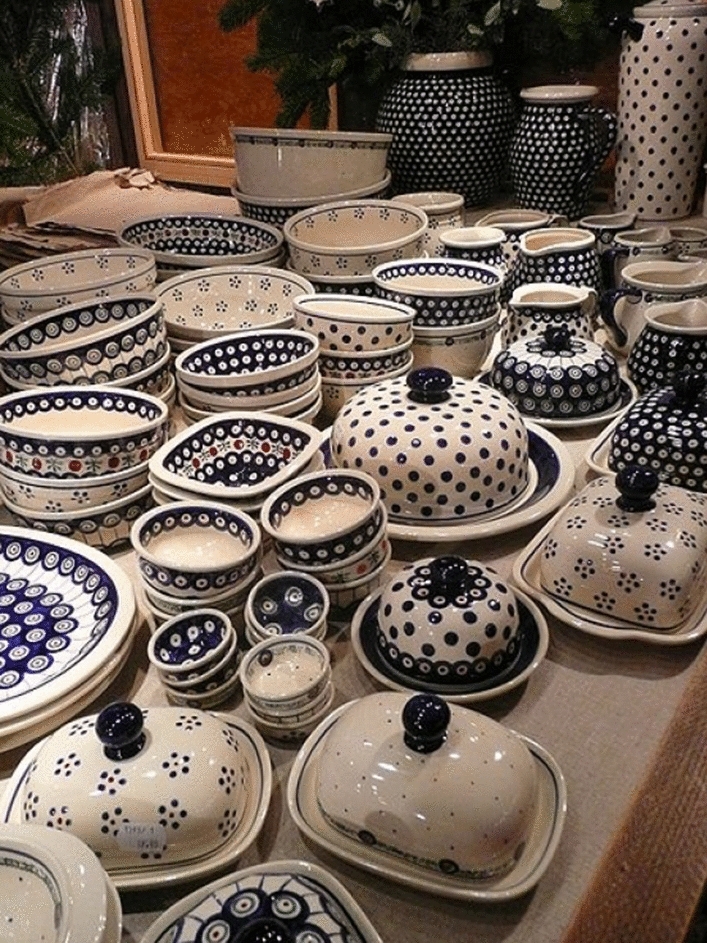
[Image of fine pottery]. (2009). Retrieved from https://commons.wikimedia.org/wiki/File:Handgefertigte_Keramik.JPG.

Our visual environment is filled with objects that vary along many feature dimensions. Most theories of vision and attention propose that attention is guided by the specific feature values that belong to the object we are looking for^1–3^. Less is known about the specific mechanisms underlying attentional guidance by features, particularly when the target object we are looking for differs from other elements in the scene along more than one visual feature. How are these featural differences combined to guide attention? Are there mechanistic laws that describe how attention is simultaneously guided by multiple features? Imagine you just finished cooking a dish and are looking for a plate to serve it. You know exactly what plate you are looking for: a white dome-like plate that has a blue-dotted pattern. Imagine searching for that specific bowl (your target) on a table that has many other plates (the distractors) that vary in shape and texture, like on Fig. 1. Does the visual system use both the overall shape and texture information to tell the target apart from the distractors? Here, we investigated the precise underlying laws that govern how texture and shape combine to guide attention in human vision during a search task.

Recent work demonstrated the existence of a mathematical law that describes how color and shape combine to guide visual attention in efficient search^4^. When observers are searching for a known target that differs from surrounding objects in terms of color and shape, the visual system computes the visual distinctiveness between the known features of the target and the distractors. Specifically, a difference signal is computed in parallel and separately for each feature. In the case of color and shape the overall distinctiveness of the target is determined by the sum of the separate color and shape distinctiveness signals. What is *visual distinctiveness* and how can it be estimated? According to Target Contrast Signal Theory^5^, visual distinctiveness reflects the difference or distance between two points in feature space, the first point being associated with the target and the second with the distractor. As described below in more details, the visual system uses visual distinctiveness signals computed throughout the visual field to reject distractors that are unlikely to be the target. In this view, Target Contrast Signal Theory^5^ therefore separates itself from theories that assume attentional guidance is driven by specific feature values^1–3^, and is more in line with theories positing attention is guided by difference signals between target and non-target items^4,6–11^. Note however that people can use peripheral vision to reject distractors^5,12–14^ only when the feature differences between the target object and the distractors are sufficiently large^12,15,16^. When the feature differences are too small, people must rely on the fine visual analysis provided by foveal vision.

**Figure 2:**
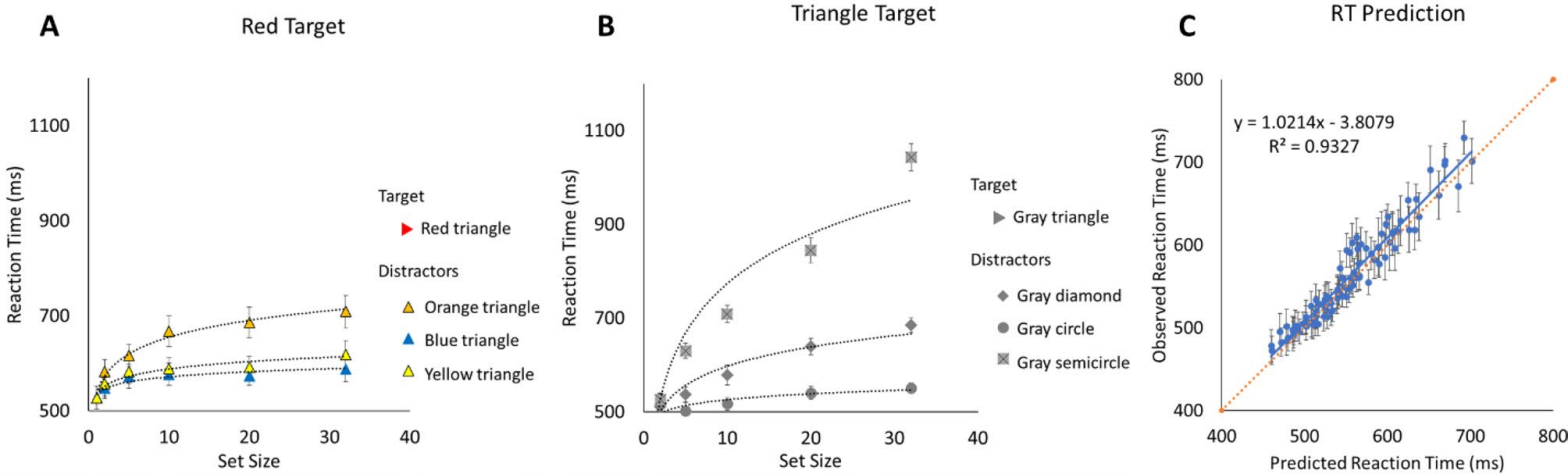
Results from Buetti et al.^4^. (**A**) Example of color search. The Figure shows reaction times when searching for a red triangle among a varying number of orange, yellow or blue triangles. Displays were always distractor homogeneous, meaning that only one distractor type was presented at a time. Results show that search efficiency, here indexed by the logarithmic search slope, varies as a function of target-distractor similarity. Search becomes less efficient when target-distractor similarity increases. (**B**) Example of shape search. The Figure shows reaction times when searching for a gray triangle among a varying number of gray diamonds, circles or semicircles. (**C**) Search efficiency observed in simple color and shape searches can be used to predict performance when the target differs from distractors both along color and shape. For instance, search efficiencies when searching for red among yellow (**A**) and when searching for triangle among diamonds (**B**) were used to predict performance when participants searched for a red triangle target among yellow diamonds. The Figure shows the predictions from the best performing model (Collinear Contrast Integration model, as is shown in Eq. (3)). Error bars on each data point indicate the standard error of the observed reaction time for each specific condition.

Visual distinctiveness can be directly derived from the observed *search efficiency* when plotting RTs as a function of set size for *each specific target-distractor feature pair*^4,5^. As shown on Fig. 2A,B, when visual search relies on parallel peripheral processing, response time increases logarithmically as a function of set size^4,5,13,14,17^. This logarithmic relationship between set size and response times is the signature that items are processed in parallel and with unlimited capacity^12,18^; in the process of finding the target, the visual system accumulates information, stochastically, in the form of a contrast signal generated when comparing an item to the target template. Once items reach a given level of accumulation (i.e., a threshold), they are rejected as potential contenders. The rate of contrast accumulation is influenced by a number of factors, such as eccentricity, but more important here, target-distractor dissimilarity. So, when the visual distinctiveness between the target and distractor is large (e.g., searching for a red target among blue items), the contrast signal will accumulate at a faster rate, resulting in shallower logarithmic slopes, that is, in faster search efficiency (blue triangle line, Fig. 2A). When the visual distinctiveness between the target and distractors is small (e.g., searching for a red target among orange items), the contrast will accumulate at a slower rate, resulting in steeper logarithmic slopes and in slower search efficiency (orange triangle line, Fig. 2A). Thus, Target Contrast Signal theory proposed that the steepness of the logarithmic slope is inversely proportional to the overall contrast signal being accumulated between a given target-distractor pairing, such that

1 $$Contrast= \frac{\alpha }{D},$$

with *D* being the logarithmic slope and $\alpha$ a multiplicative constant.

As an example, to determine the visual distinctiveness (referred to as Contrast_overall_ in Eq. (2)) of a target that differs from distractors along two separate features, say a red triangle among yellow diamonds, one needs to determine the visual distinctiveness along the color feature space and along the shape feature space. That is, logarithmic search efficiencies must be obtained when searching for a red triangle target among yellow triangle distractors (color search; Fig. 2A) and when searching for a gray triangle target among gray diamond distractors (shape search; Fig. 2B). Buetti et al.^4^ tested different equations to determine how the contrast along color and the contrast along shape should be combined and found that Eq. (2) was the best formula to determine the overall visual distinctiveness between target and distractors when the stimuli differed along color and shape. Given that the visual distinctiveness is related to the logarithmic search slope as indicated in Eq. (1), if one replaces Eqs. (1) into Eq. (2), Eq. (3) and (4) obtain, with ${D}_{color}$ being the logarithmic slope observed in color search for a specific target-distract pairing and ${D}_{shape}$ being the logarithmic slope observed in shape search for a specific target-distractor pairing. Note that the $\alpha$ cancels out on both sides of the equation when one replaces Eq. (1) into Eq. (2).

2 $${Contrast}_{overall}={Contrast}_{color}+ {Contrast}_{shape,}$$

3 $$\frac{1}{D}_{overall}= \frac{1}{D}_{color}+ \frac{1}{D}_{shape},$$

4 $$\mathrm{which\, solves\, to} {D}_{overall}= \frac{{D}_{color} \times { D}_{shape}}{{D}_{color} + {D}_{shape}}.$$

The measure of visual distinctiveness provided by *D*_*overall*_ (Eq. (4)) can then be used to *predict search times* in different, more complex, search conditions using Eq. (5).

5 $${RT}_{predicted}={RT}_{0}+{D}_{\mathit{overall}\mathit{ }}\mathrm{* ln}\left(setsize\right).$$

In Eq. (5), RT_0_ corresponds to the response time in the target only condition, ${D}_{overall}$ corresponds to the predicted overall search efficiency computed from Eq. (4) for the combined contrast of color and shape. The final term is the natural logarithm of the total set size, which includes all distractors plus the target.

Buetti et al.^4^ used visual distinctiveness measures observed for specific target-distractors color-parings (Fig. 2A) and for specific shape-parings (Fig. 2B) to predict 90 search times when the target differed from distractors along both color and shape features. The predictions from Eq. (5) were then compared to the observed search times from six experiments in which separate groups of naïve participants searched for one of two targets (a red triangle or a blue cyan semicircle) among a set of homogeneous distractors varying both in color (e.g., orange, blue, or yellow) and shape (e.g., diamonds, circles, triangles). Remarkably, as shown on Fig. 2C, Eq. (5) accounted for 93.3% of the variance observed in the data with a mean prediction error of only 13 ms (the corresponding data and code are available on OSF, link: https://osf.io/f3m24/).

### Texture as a visual feature

A question that follows Buetti et al.’s^4^ work is how does this finding extend to other visual features? We decided to investigate how texture and shape combine because in our daily life we often encounter objects (i.e., shapes) that are characterized both by given colors but also by specific patterns or surface textures (Fig. 1). Thus, investigating how shapes and texture combined seemed a natural way of pursuing the investigation of how features combine. The literature provides some information on this front. Admittedly, this type of surface property is not the only type of texture that could be studied. In the Supplementary Materials, section we motivate why we chose this kind of texture as opposed to other ones like material texture, which has also been studied^19–21^.

Garner and Felfoldy^22,23^ determined that there are two types of relationships between feature dimensions: integral and separable. To evaluate the relationship the authors used a speed classification task. Participants were shown stimuli on cards that varied along two dimensions, say length and width, and were asked to sort the cards into two categories (e.g., long vs. short) by focusing only on one dimension (e.g., length). Participants were asked to ignore the other dimension (e.g., width). If the irrelevant dimension affected the speed at which participants sorted the relevant dimension, the two dimensions were defined as *integral*. If no such interference was observed, the two dimensions were called *separable*. Garner^22^ showed that the dissimilarity along separable feature dimensions (e.g., color and shape^4,24^) combine linearly, following a city-block metric; on the other hand, dissimilarity along integral feature dimensions (e.g., saturation and brightness of Munsell colors^23^) combine following a Euclidean distance metric. Note that Garner’s City block metric corresponds to Eq. (2) and Garner’s Euclidian distance metric corresponds to Eq. (7) below.

Later works inspired by Garner’s findings tested other feature combinations using the same speed classification task and showed inconclusive evidence regarding texture and shape. Cant et al.^19^ found that neither material texture (brick or wood textures) or surface color interfered with the width or length classification. Meiran et al.^25^ found that surface texture (dots or lines) was separable from shape as well as from color. Supporting these findings, recent fMRI and neuropsychological data provided evidence that different cortical structures are responsible for the processing of shape (lateral occipital cortex) and material properties (collateral sulcus), suggesting that these properties are independently coded^20^. However, there is also evidence that other texture information (such as curved or straight lines that made the surface appeared convex or flat), interfered with shape classification^26^, suggesting these features are integral.

Texture as a visual feature has been mostly studied with regards to the process of texture segmentation^27–33^, that is, the study of the conditions under which the visual system can effortlessly or “pre-attentively” separate two regions of the scene based on textural differences. These regions are constructed by repeating elements and the question is which featural characteristics of those repeated elements afford segmentation in a parallel fashion without requiring serial attention to individual elements. Julesz^27,28^ showed that texture segmentation can happen effortlessly when the repeating elements differ along color, elongated blobs (i.e., line segments with different orientations and widths), and terminators (the end-points of the elongated blobs). Differences in size, and contrast can also afford texture segmentation^30^. More generally, texture segmentation is thought to arise from analysis of *global* display properties, that is, properties shared by many items, that differ across different regions of the display and that may be unavailable at the single item level^34^. For instance, the average orientation of lines in one region of the display may differ from the average orientation of lines around that region, creating a segmentation clue.

The role the textural properties of an object play in directing attention towards objects that share those same properties has seldom been studied, aside from color. That is, we know that color is a powerful guiding feature^35^, but if we go beyond color to the shape patterns that may exist on the surface of objects, it is less clear how well the human visual system can analyze that information and use it to guide attention in a scene. One might intuit that if a shape pattern affords segmentation when embedded in a second shape pattern, then visual search for objects with that shape pattern ought to be parallel and effortless, and vice-versa. But this is not the case^34^. There are shape patterns that can afford effortless segmentation without giving rise to parallel search. The reverse is also true. There are shape patterns that can be found in parallel but nonetheless do not afford effortless segmentation. According to Wolfe^34^, the reason for this is that segmentation follows analysis of global properties of the scene, whereas search is focused on properties of individual objects in that scene. In fact, when there is only one target in the display, the availability of its visual properties as “global” properties of the display is vastly diminished, compared to when multiple identical items share that same property in close proximity of one another (as in texture segmentation).

More recently and related to the current study, Pramod and Arun^21^ studied how material texture and shape combine in efficient visual search. The authors focused on the inverse of reaction time (1/RT) at a fixed set size, as a measure of the distance between target and distractors in feature space^36^, that is, as a measure of target-distractor dissimilarity. A weighted linear combination model was used to compute the expected 1/RT score in a bi-dimensional search condition (when the target differed from distractors along both texture and shape) based on the 1/RT scores in the unidimensional searches. The results showed that the unidimensional search parameters for texture and shape were sufficient to account for 83% of the variance observed in bidimensional search displays. Although successful, the methodology has some shortcomings. Because the parameters are estimated at a specific set size, the fitted model is only capable of interpreting the data at this fixed set size; this harms the generalizability of the model because the dissimilarity score between the same two stimuli is different for different levels of set size. Further, the 1/RT measure compounds search-related processing time with non-search processing time, such as response selection and execution, which contaminates the dissimilarity metric and makes it less accurate in reflecting the searching process. Finally, the authors only used weighted linear combinations in their formulas and did not include comparison to other non-linear metrics like the Euclidian distance metric^22^.

### Present study: how do surface texture and shape combine to guide attention?

The present study follows the prediction-based methodology of Buetti et al.^4^, which consists of three steps.

#### Step 1: estimating search efficiency in one-feature dimension search

**Figure 3:**
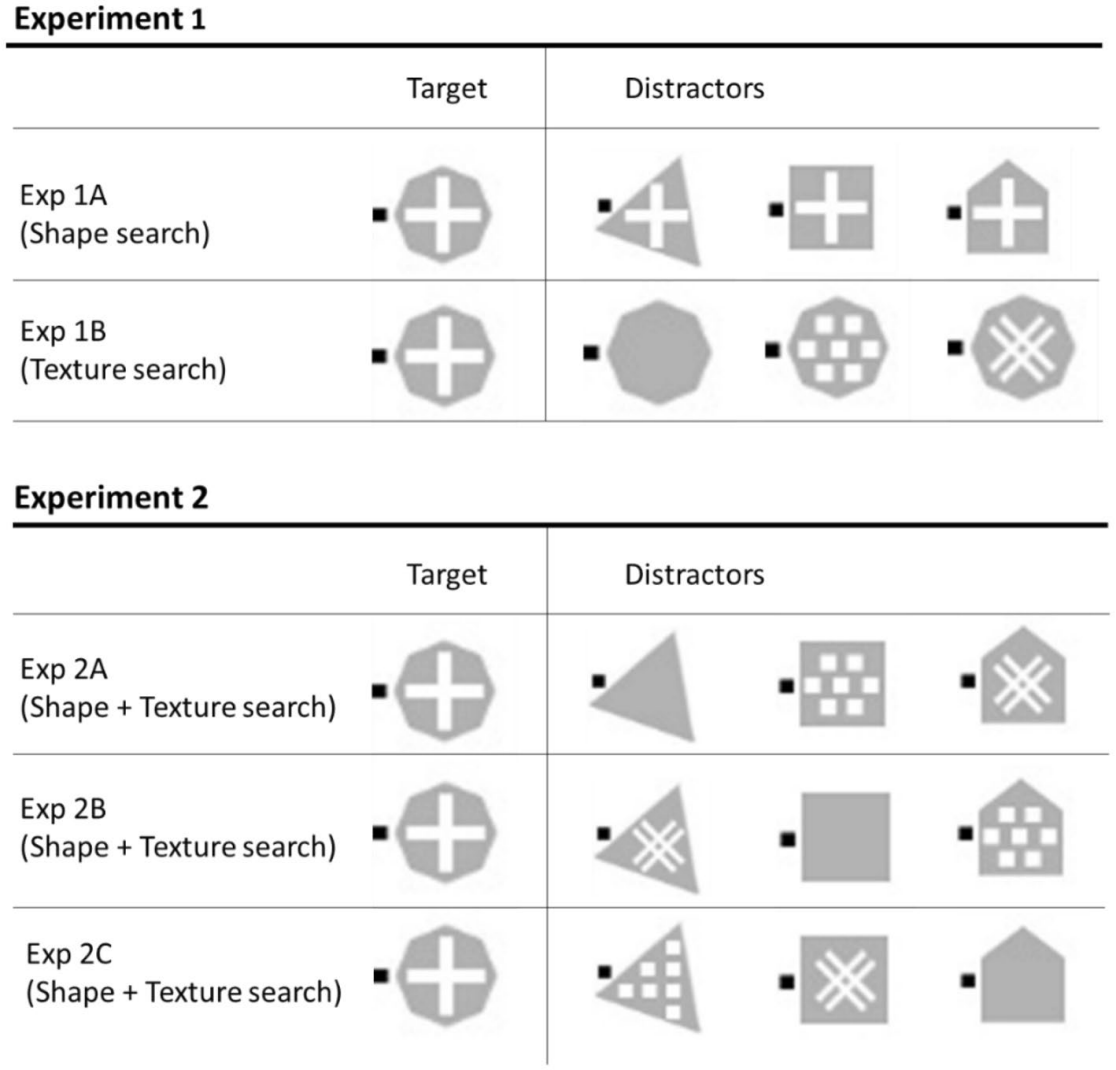
Stimuli used in Experiments 1 and 2. The black squares near stimuli were presented either on the left or right of the stimulus. Participants were asked to report the left or right location of the black square of the target item. A group of naïve participants completed each experiment.

In Experiment 1, the target and distractors differed along one single feature, either shape or texture. The goal of the experiment was to estimate the logarithmic search efficiency (i.e., the *D* values) for all target-distractor pairings shown in Fig. 3^37^. These *D* values will then be used to predict search times when the target differs from distractors along two features, shape and texture, in Experiment 2.

**Figure 4:**
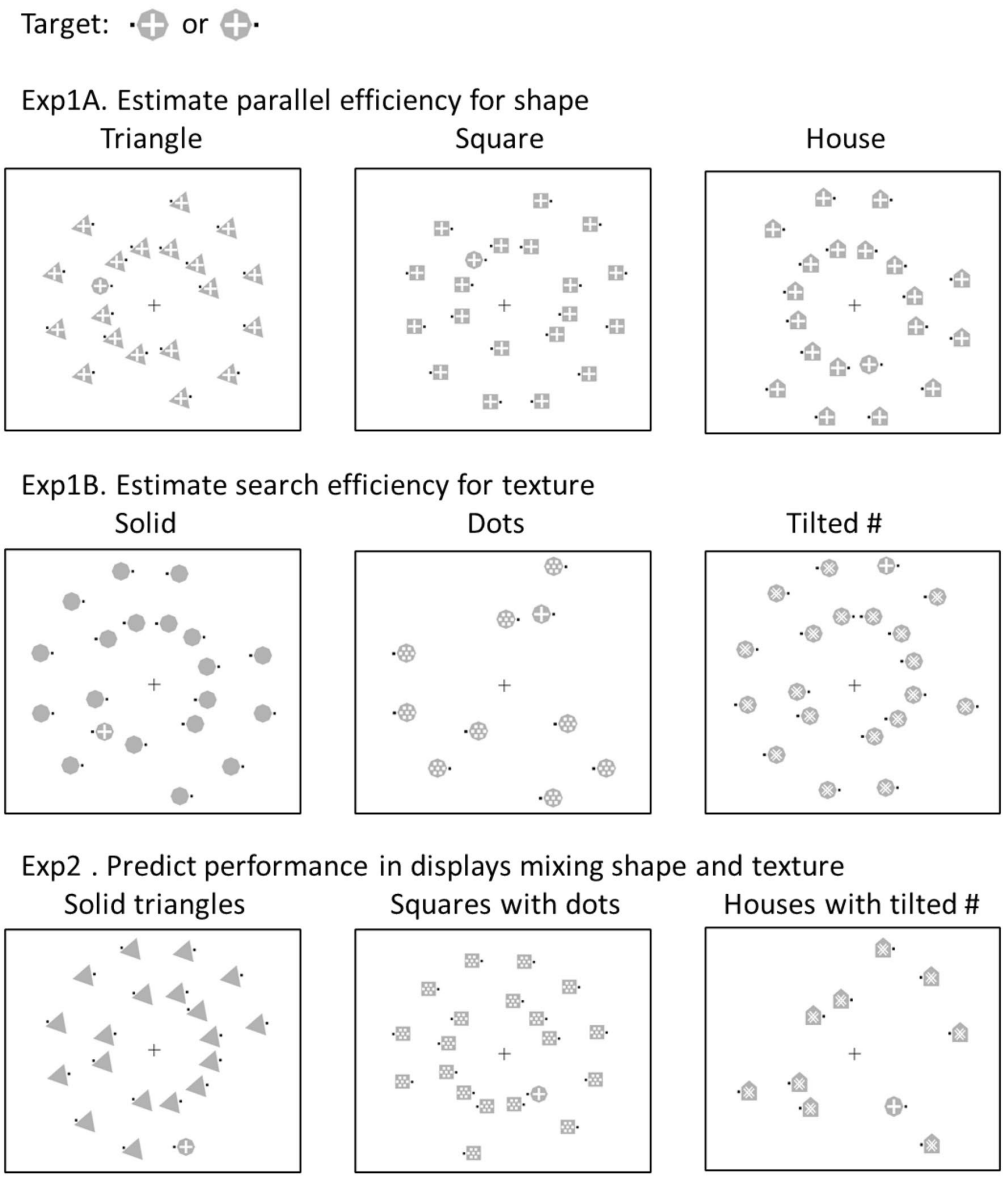
Illustration of the approach used in the present study. The target was always an octagon with a white cross texture. In Experiment 1A, in the shape search condition, logarithmic search efficiency was evaluated when participants searched for the target among 0, 1, 4, 9, and 19 identical distractors (either triangles, squares or houses). The distractors shared the same white cross texture as the target. In Experiment 1B, in the texture search condition, logarithmic search efficiency was evaluated when participants searched for the target among 0, 1, 4, 9, and 19 identical distractors (octagons with either a tilted pound key, dotted or solid texture). In Experiment 2, in the combined shape and texture conditions, logarithmic search efficiency was evaluated when participants searched for the target (octagon with white-cross texture) among distractors that differed along the shapes and textures tested in Experiment 1A and B. In Experiments 2A–C, search performance was evaluated for all combinations of shape (3) and texture (3) distractors, as shown on Fig. 3 (bottom).

In all experiments, the target was a gray octagon with a white cross and participants reported the left or right location of the black square next to it. In Experiment 1A, the search was based on *shape*: the distractors shared the same texture as the target, a white cross on a gray background, but differed in shape; they were either triangles, squares, or houses (Fig. 4 top). In Experiment 1B, the search was based on surface *texture*: the distractors shared the same shape as the target, a gray octagon, but varied in texture; the textures were either white dots, or white lines forming a tilted pound key, or solid gray (Fig. 4 middle).

#### Step 2: estimating search efficiency in two-feature dimensions search

In Experiment 2, search times were evaluated for all possible *combinations of surface texture and shape distractors*. Three groups of naïve participants completed Experiment 2A–C. Each group of participants searched for the same target among three different types of distractors (Fig. 3 bottom). Displays contained only one type of distractors (Fig. 4 bottom).

#### Step 3: model comparison: predicting search RTs observed in Step 2 by using the parameters observed in Step 1

The logarithmic slopes for each specific shape and texture pairings from Experiment 1A and 1B were then used to predict the logarithmic slopes (i.e., the *D*_*overall*_ values to be used in Eq. (5)) when the target differed from distractors both in terms of shape and texture. The *D*_*overall*_ values were computed for three different models described in Eqs. (6)–(8). For each model, the *D*_*overall*_ values were used to predict the 36 search times using Eq. (5) (i.e., 9 target-distractor pairings by 4 distractor set sizes). These predicted search times were then compared to the observed search times from Experiment 2A–C. The predictive performance of each model was then compared. We also considered a fourth model based on Pramod and Arun’s^21^ work that used the 1/RT index instead of the *D* index (Eq. (9)).

**Figure 5:**
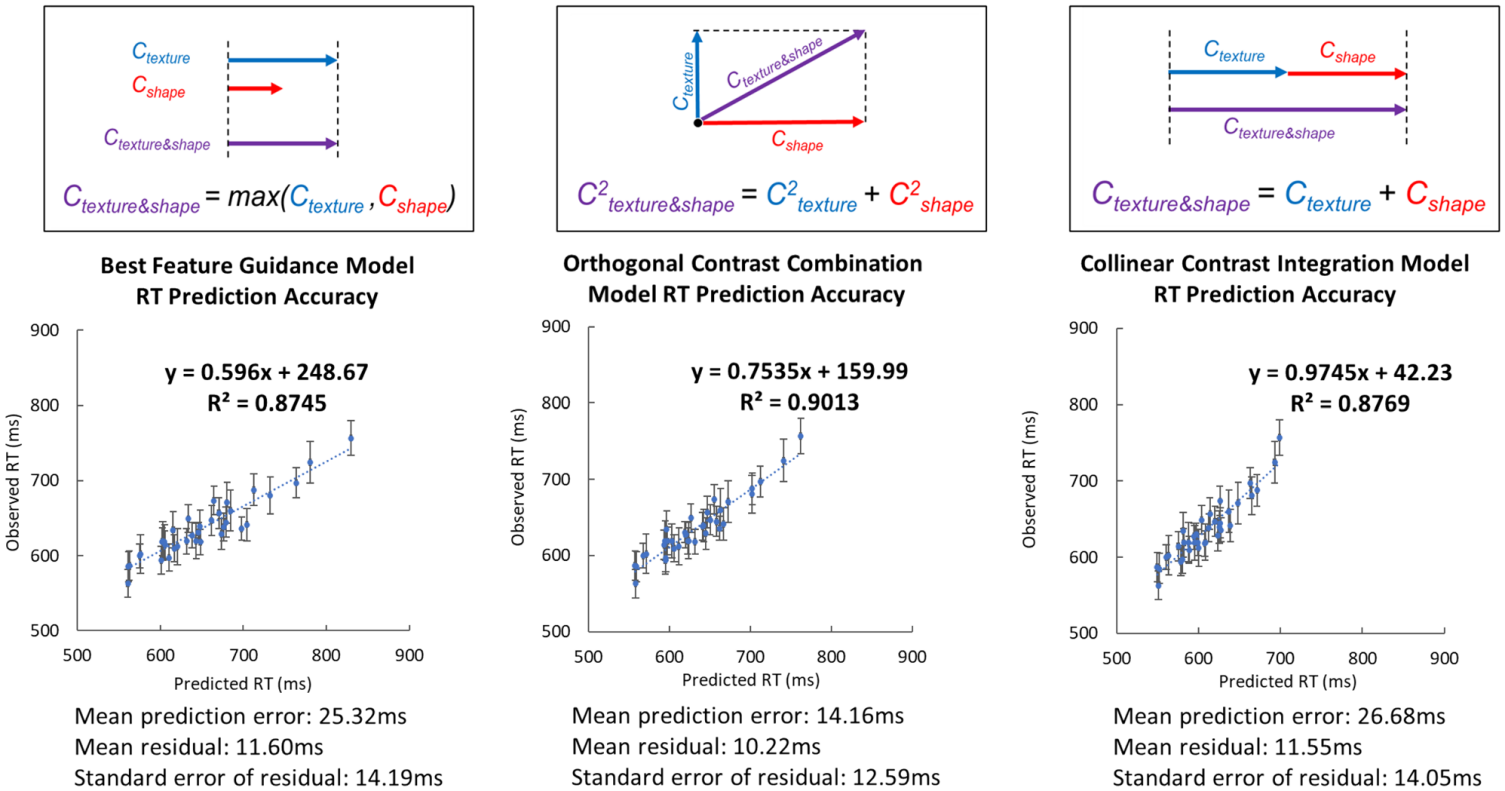
*Top* Visualization of how contrasts along texture (*C*_*texture*_) and along shape (*C*_*shape*_) could be combined to produce the overall contrast when a target differs from distractors along two feature dimensions (*C*_*texture*&*shape*_) according to the Best Feature Guidance Model (left panel), the Orthogonal Contrast Combination Model (middle panel), and the Collinear Contrast Integration Model (right panel), respectively. *Bottom* Observed RTs from Experiment 2A–C as a function of Predicted RTs. Predicted RTs were estimated using Eq. (5). The left, middle, and right panels show the prediction accuracy of Eq. (5) when the D_*overall*_ parameter was computed based on the Best Feature Guidance Model (Eq. (6)), the Orthogonal Contrast Combination Model (Eq. (7)), and the Collinear Contrast Integration Model (Eq. (8)), respectively. Error bars on each data point indicate the standard error of the observed reaction time for each specific condition.

The equations for the four models are described below:

1. *Best feature guidance model* This first model assumes that when the target and the distractors differ in both shape and texture, the visual system will rely on the dimension that provides the largest contrast (Eq. (6)), indexed by the smaller *D* value. The visual system will ignore the contrast coming from the other feature (Fig. 5 top left panel).
  6 $${D}_{overall}=\mathrm{min} \left({D}_{texture }{, D}_{shape}\right).$$
2. *Orthogonal contrast combination model* If shape and texture are integral dimensions, then the two dimensions should combine according to Garner’s Euclidian distance metric^4,22^. According to this model, the overall contrast is formed in a shared multidimensional space composed by the single feature dimensions of shape and of texture (Fig. 5 top middle panel). The magnitude of the overall contrast is determined by the orthogonal sum of the two single-feature contrasts (i.e., the Euclidean distance), both computed in an independent manner. The overall contrast would be expressed like: *Contrast*_*overall*_^2^ = *Constrast*_*texture*_^2^ + *Constrast*_*shape*_^2^. Since the overall contrast for a given target-distractor pairing is inversely proportional to the steepness of the logarithmic slope *D*, this formula solves as follows:
  7 $${D}_{overall}= \frac{1}{\sqrt{{\left(\frac{1}{{D}_{texture}}\right)}^{2}+{\left(\frac{1}{{D}_{shape}}\right)}^{2}}}$$
3. *Collinear contrast integration model* This model assumes independence of the two feature dimensions (similar to the Orthogonal Combination Model) but the two contrasts are combined collinearly (Fig. 5 top right panel). This means that the two contrast are maintained separately and are not combined in a common multidimensional space to produce the overall contrast. In Garner’s terms, the Collinear Contrast Integration Model follows a the city-block distance metric^22^ such that: *C*_*overall*_ = *C*_*texture*_ + *C*_*shape*_. Since the contrast is inversely proportional to *D*, the formula can be solved as:
  8 $$\frac{1}{{D}_{overall}}= \frac{1}{{D}_{texture}}+ \frac{1}{{D}_{shape}}.$$
  Note that Eq. (8) is the same as Eq. (3) and is the equation that was found to best predict how color and shape combine to guide attention in Buetti et al.^4^.
4. *Reciprocal of RT model* This model is similar to the Collinear Contrast Integration Model but uses a different metric to predict feature combinations. Specifically, instead of using the search slope estimated across all set sizes like Buetti et al.^4^, the model uses *1/RT at a fixed set size as an index of target–distractor feature distance*. Pramod and Arun^21^ used set size 16. Here we used set size 20.
  9 $${1/RT}_{s,t}=a\times {1/RT}_{s}+b\times {1/RT}_{t}+c.$$

In Eq. (9), a, b, and c are free parameters that are optimized to minimize residual error. $1/RT$_*s*_ represents the 1/RT measured when target and distractors differed along shape only; $1/RT$_*t*_ represents the 1/RT measured when target and distractors differ along texture only; $1/RT$_*s,t*_ represents the 1/RT measured when the target and distractors differed along both shape and texture.

## Results

### Step 1: search efficiency in one-feature dimension search

Table 1 shows the logarithmic slopes as well as the RTs at set size 20 found in Experiments 1A, B. These parameters were used to make RT predictions in Step 3 below.

**Table 1:** Logarithmic search efficiency (*D*) in Experiments 1A and 1B as well as reaction times (RTs) at set size 20.

Stimuli	Distractor type	D	RTs (set size = 20)
Shape	House	95.2	846.7
	Square	44.9	693.2
	Triangle	46.8	689.6
Texture	Solid	43.9	693.2
	Tilted#	111.9	905.8
	Dots	69.5	766.1

### Step 2: search efficiency in two-feature dimensions search

Table 2 shows the logarithmic slopes as well as the RTs at set size 20 observed in Experiments 2A–C.

**Table 2:** Logarithmic search efficiency (*D*) in Experiments 2A–C as well as reaction times (RTs) at set size 20.

Compound stimuli	Distractor shape	Distractor texture	*D*	RTs (set size = 20)
Experiment 2A	House	Tilted#	68.6	756.9
	Square	Dots	28.2	644.8
	Triangle	Solid	29.1	638.5
Experiment 2B	House	Dots	49.1	724.7
	Square	Solid	19.2	641.6
	Triangle	Tilted#	37.0	687.8
Experiment 2C	House	Solid	38.1	647.0
	Square	Tilted#	46.1	673.6
	Triangle	Dots	38.7	657.1

### Step 3: model comparison

Figure 5 shows the observed RTs from Experiment 2A–C as a function of predicted RTs. As a reminder, predicted RTs were computed using Eq. (5). The parameter D_*overall*_ in Eq. (5) was computed based on the Best Feature Guidance Model (Eq. (6)), the Orthogonal Contrast Combination Model (Eq. (7)), and the Collinear Contrast Integration Model (Eq. (8)). Equation (5) therefore produced a precise RT for each of the set size tested in Experiments 2A–C.

The results indicated that the R^2^ of the Orthogonal Contrast Combination Model (90.13%) was higher than the R^2^ of the Best Feature Guidance (87.45%) and Collinear Contrast Integration (87.69%) models. The AIC model comparison results showed that the Orthogonal Contrast Combination Model (AIC = 288.45) was 75 times more likely than the Best Feature Guidance Model (AIC = 297.10) and 53 times more likely the Collinear Contrast Integration Model (AIC = 296.39).

Furthermore, we computed the mean prediction error to evaluate the accuracy of the predictions of each model. The mean prediction error was obtained by computing the mean of the absolute differences between the observed RTs in Experiments 2A–C and the RTs predicted by each model using Eq. (5). Larger values indicate that the predicted RTs are scattered more widely around the observed values. Smaller values indicate that the predicted RTs are more tightly clustered around the observed values. The Orthogonal Contrast Combination Model showed a smaller prediction error (14.16 ms) than the Best Feature Guidance Model (25.32 ms), t(35) = 3.41, p = 0.002, dz = 0.824, BF = 20.17, and than the Collinear Contrast Integration Model (26.68 ms), t(35) = 5.41, p < 0.001, dz = 1.307, BF = 4200.37. Note that the prediction range was about 200 ms (observed RT range 563–757 ms), so the magnitude of the mean prediction error of the winning model was about 7% of the overall prediction range. In fact, 35 of the 36 predictions of the winning model (Orthogonal Contrast Combination Model) fell within the margin of error of the observed data (i.e., inside an interval centered on the observed mean plus or minus 1.96 times the standard error of the observed mean).

Finally, the comparison between the winning model and the Reciprocal of RT Model indicated that the R^2^ of the Orthogonal Contrast Combination Model (90.63%) was higher than the R^2^ of the Reciprocal of RT Model (85.13%). We also computed the AIC relative likelihood of the two models, and the results showed that the Orthogonal Contrast Combination Model (AIC = 76.25) was 8 times more likely than the Reciprocal of RT Model (AIC = 80.41).

We should add we had started a replication of the current results, but data collection was stopped abruptly due to the COVID-19 pandemic. The partial data are reported in the methods section and confirm that Orthogonal Contrast Combination outperforms the other two models.

## Discussion

Visual objects can differ from one another along a multitude of visual properties. Although it has been clear for a long time that certain features can by themselves guide attention in a scene^35^, the question of how differences along multiple feature dimensions each contribute to guide attention has not been the focus of much research, particularly when the observers know ahead of time which features they are looking for. Buetti et al.^4^ showed that for color and shape the visual system is computing visual distinctiveness values for each feature dimension separately, which are then combined linearly to guide attention. The present study investigated two different feature dimensions: shape and surface texture. Several conclusions can be drawn from the present results.

First, the results indicated that textural information alone can guide attention in parallel across the scene, indicating that peripheral vision can compute visual distinctiveness signals arising from the surface properties of objects. This follows because the RT by set size functions in Experiment 1B were logarithmic (data shown in Supplementary Information Fig. S1), a behavioral index of parallel guidance across the scene^4,5,12^. In sum, this demonstrates for the first time that other surface properties beside color^35^ can guide attention, specifically, shape-defined textural elements present on the surface of objects.

Second, the winning model combined surface texture and shape in orthogonal fashion, suggesting that surface texture and shape combine following a Euclidian metric and are integral dimensions^22^. This finding makes sense if one considers the surface textures used in the present study as shapes within shapes. In that sense, the stimuli here can be understood as hierarchical stimuli, much like Navon letters: they each have a global feature property (i.e., the overall shape of the object) and a local feature property (i.e., the smaller shapes inside the object boundaries). The global feature (shape) would be carried over low spatial frequency channels and the local feature (texture) would be carried over relatively higher spatial frequency channels but importantly, both would rely on the same brain areas for processing. When viewed from this perspective, the finding that both the global and local features used here concurrently guide attention is consistent with Miller’s^38^ proposal that, more generally speaking, local and global properties of objects are processed in parallel and are available at the same time to guide attention.

Third, the result showed that when searching for a target that differs from distractors along two visual features, both features contribute to attentional guidance, irrespective of their efficiency. It is not the case that the feature dimension with the greatest guidance will drive performance, as evidenced by the finding that the Best Feature Guidance model was outperformed by the Orthogonal Contrast Integration Model which takes into consideration differences along both feature dimensions. Thus, this suggests that when processing information in parallel, the visual system uses *all* available distinctiveness signals to guide attention. This observation is consistent with Buetti et al.’s^4^ findings showing that shape contributed to guidance even though it was systematically less efficient than color. An implication of this finding is that the more information an observer has about the visual properties of a target, the more efficient the observer will be at finding that target. This is consistent with previous findings showing that the more precise the target-defining cue is (verbal label vs. categorical example vs. exact image of the target), the more efficient the search becomes^39,40^. These findings are also consistent with the “information summation” proposal^41^. In that study, the authors studied whether observers rely on contour cues and/or surface texture cues (composed of small Gabor elements) when detecting the presence of a large shape in a field of randomly oriented Gabors. The authors found that observers simultaneously used both cues in a manner that allowed them to perform better than if they had relied on either of the two cues, or even on the best of both cues. This indicated that information about contour and surface texture was combined in the visual system (much like in Eq. (8)) to improve the detectability of an object in noise.

Fourth, the results suggest that texture and shape co-activate to guide attention. This can be concluded from the fact that the slope of the Observed by Predicted RT function was 0.75 (Fig. 5 middle). Because the slope is smaller than 1, it indicates that the presence of distinctiveness signals along shape and texture *speed up attentional guidance* in a multiplicative way. Specifically, the observed RTs when people are searching for a target that differs from the distractors along both texture and shape was 25% faster than the simple Euclidian summation of the shape and texture distinctiveness signals.

Fifth, it should also be noted that the findings showing that texture and shape combine according to a Euclidian metric stand in contrast with previous findings suggesting that shape and texture are separable features^19,25^ that are independently coded in the brain^20^. We suggest this inconsistency may arise from the type of visual processing used to perform the tasks. For example, the speed-classification task^19,25^ relies on foveal vision. Similarly, the oddball detection task^20^ also relies on foveal vision (observers were free to foveate the stimuli over long periods of time to decide which of three shapes was different from the other two along either shape or material texture properties). Thus, it is possible that in tasks where only one object at a time is examined with foveal vision, observers might be able to maintain the representation of the two feature dimensions separately (giving rise to evidence for separable dimensions). In contrast, in a parallel search task where multiple objects are presented in the periphery, observers might no longer be able to maintain distinct representations. In this case, the representations of texture and shape might be combined into a single dimensional feature space that characterizes each object at a given location. This would occur because of the properties of peripheral vision. Indeed, peripheral vision tends to represent information in a pooled manner, where visual information at a given location is pooled into a summary representation of the information at that location^15^, as opposed to the detailed representations afforded by foveal vision. Therefore, when studying how two or more features combine, it seems important to consider not only the specific features in question but also the type of visual processing (foveal vs. peripheral) involved in representing those features. These findings further suggest that while certain feature combinations are pooled (like shape and texture, as shown here), other feature combinations (like shape and color, see Buetti et al.^4^) appear to survive pooling, as also shown in the visual crowding literature^42–44^.

Finally, the current study represents a new demonstration of the success of our estimate-then-predict methodology^4,45–47^. This approach has also been used to investigate other theoretical questions such as why search slows down when multiple types of distractors are intermixed in the same display (distractor heterogeneity effect)^45–47^. This success is owed to two factors: (1) Equation (5) captures the processes at play in peripheral parallel search, specifically, that the rejection of non-target items in peripheral vision follows a logarithmic efficiency; (2) the estimates of the *D* parameters indexing visual distinctiveness are robust across experiments and across groups of observers, underscoring the proposal that feature distinctiveness or contrast is the currency of attentional guidance^5^. The manner in which these distinctiveness signals for each feature are combined to determine the overall object’s contrast to the target item seem to follow different laws. Distinctiveness signals along color and shape dimensions combine linearly^4^, while signals along surface texture and shape combine orthogonally, likely reflecting the differences in the way the visual system represents those feature dimensions in the first place. This critical idea behind Target Contrast Signal theory that contrast is the main currency of the visual system and the force that guides attention makes sense from a neurobiological perspective. Most of the visual brain is wired to compute contrasts. For instance, most color-coding neurons in the early visual system code color contrasts as opposed to specific color values and are present as early as the LGN^48,49^, in fact, they are computed even before visual information leaves the eyeball^50^. A contrast computation is also at the basis of all lateral inhibition signals^51^, of the color system^52^, of boundary detection^53^, and perhaps even of visual categorization^54^. This idea that the contrast or difference between two stimuli is critical for determining search performance is also consistent with evidence from neuroscience showing search speed is proportional to the discriminability between patterns of neuronal activity in visual cortex that respond to target and distractors^55–57^. Perhaps theories of visual attention should shift the focus away from arguing that specific feature values guide attention^1,2,35,58,59^, and towards a more relational consideration, where attention is guided by the context in which features present themselves, as proposed by Target Contrast Signal theory^5^ and the relational account of attentional guidance^6–11^.

## Methods

The methods and experimental protocols were approved by the Institutional Review Board at the University of Illinois, Urbana-Champaign, and are in accordance with the Declaration of Helsinki.

The present study uses a model comparison approach to study visual attention that side steps the traditional null hypothesis significance testing. The goal is to find a theoretically motivated model that accounts for the most variance in the data by making specific reaction time predictions for the different experimental conditions. A total of five experiments were conducted (Experiment 1A, 1B, 2A, 2B and 2C), each with a naive group of participants. The data and code from this project are publicly available on OSF (https://osf.io/pkh68/).

### Participants

Participants were recruited from the University of Illinois at Urbana Champaign and participated in the experiments in exchange for course credit or pay ($8 per hour). Informed consent was obtained from all participants. Sample size was determined based on previous studies in our lab showing that averaging the data of twenty subjects produces stable estimates of the group means of reaction times and search slopes for a given search condition^12–14,17^, and is sufficient to obtain substantial differentiation between models^4,45–47^. For each experiment, two inclusion criteria were used: search accuracy should be higher than 90% and the individual’s average response time should fall within two standard deviations of the group average response time. Data collection was stopped as soon as 20 participants met these two criteria. As a result, 20 and 21 participants participated in Experiment 1A and 1B, respectively; 21, 21 and 20 participants participated in Experiment 2A, 2B and 2C, respectively. One participant from Experiment 1B (group accuracy = 0.99, mean RT = 691.25 ms, sd = 106.92), one participant from Experiment 2A (group accuracy = 0.98, mean RT = 631.53 ms, sd = 76.03), and one participant from Experiment 2B (group accuracy = 0.99, mean RT = 644.45 ms, sd = 101.12) were excluded because they did not meet the response time criterion. No participant was excluded from Experiments 1A (group accuracy = 0.98, mean RT = 662.07 ms, sd = 109.94) and Experiment 2C (group accuracy = 0.99, mean RT = 612.70 ms, sd = 80.02).

**Figure 6:**
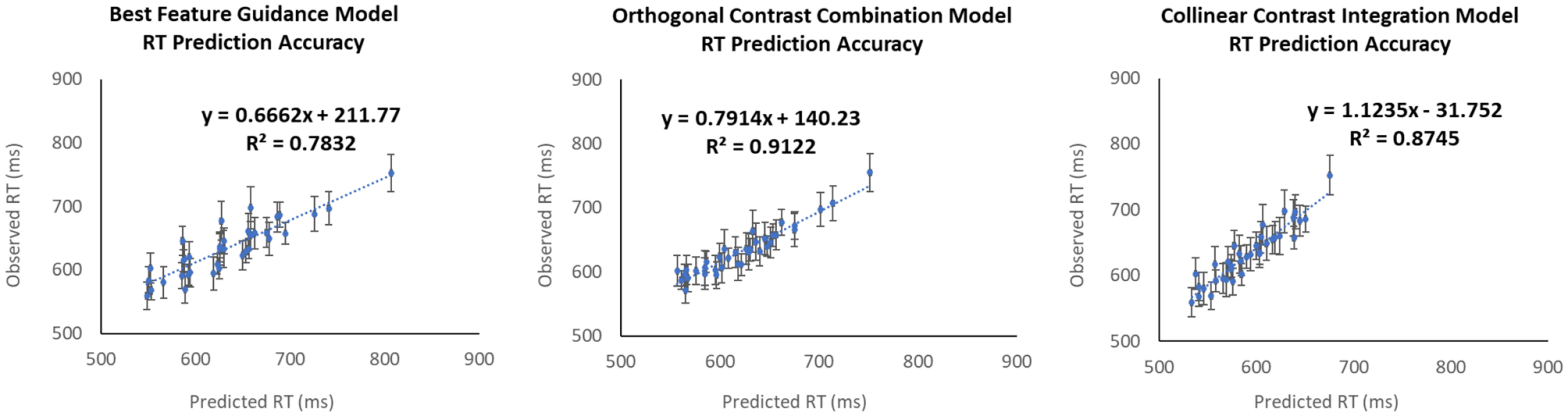
Reaction time prediction accuracy of Eqs. (6)–(8) according to the Best Feature Guidance Model (left panel), the Orthogonal Contrast Combination Model (middle panel), and the Collinear Contrast Integration Model (right panel), respectively, based on the unfinished confirmatory study. Error bars on each data point indicate the standard error of the observed reaction time for each specific condition. Once again, the Orthogonal Combination Model outperformed the other two models.

Note that the present study was pre-registered on OSF (link: https://osf.io/br965). The goal of the pre-registration was to propose to re-run Experiments 2A–C with new groups of naïve participants to confirm the results of the model comparison in a separate independent sample. In the pre-registration, we proposed to run 35 participants in each group in the confirmatory study to improve the precision of RT estimates. Unfortunately, due to the onset of the Covid-19 pandemic, all data collection at our institution was halted. At that time, we had collected a total of 16, 19, and 8 participants in the three two-feature dimensions experiments. The results from this partial sample are shown in Fig. 6, as a comparison.

### Apparatus and stimuli

All experiments were programmed in Matlab using Psychophysics Toolbox 3.0 extension and run on 64-bit Windows7 PCs. All stimuli were presented on a 20-CRT monitors at an 85 Hz refresh rate and with a 1024 × 768 resolution. Stimuli were about 45 × 45 pixels in size, 1.88 × 1.88 degrees in visual angle, randomly assigned to a location on the display based on two circular grids centered at the center of the screen. The larger grid had a diameter of 557 pixels (22.74 degrees in visual angle), and the smaller grid had a diameter of 300 pixels (12.39 degrees in visual angle). All the stimuli were gray, shown on a white background. Only one type of distractors was shown on the display on a given trial (i.e., displays were homogeneous). In all experiments, the stimuli had a black square on either the left or right side of the shape and participants were asked to report the location of the square on the target stimulus.

In Experiment 1A (shape search), the stimuli shared the same texture, a white cross on a grey background. The stimuli varied in their shape: the target was an octagon and the distractors were either triangles, squares, or houses.

In Experiment 1B (texture search), the stimuli shared the same shape, a grey octagon but differed in terms of the texture included in the shape. The target had a white cross and the distractors had either white dots, white lines forming a tilted pound key, or a solid gray texture.

In Experiment 2A–C, the target was an octagon with the white cross on a gray background. In Experiment 2A, the distractors were either squares with white dots, houses with a tilted pound key, or triangles with a solid gray background. In Experiment 2B, the distractors were either houses with white dots, triangles with a tilted pound key, or squares with a solid gray background. In Experiment 2C, the distractors were either triangles with white dots, squares with a tilted pound key, or houses with a solid gray background.

### Design

In all five experiments (Experiments 1A, B and 2A, C), there was a target-only condition where no distractors were presented. For each type of distractors, there were four distractor set sizes: 1, 4, 9, 19. In each experiment, three types of distractors were tested. In total, each experiment contained 13 conditions that were repeated 48 times, summing up to a total of 624 trials.

### Procedure

Each trial began with a black cross appearing for one second at the center of the screen over a white background. A search display followed. Participants were asked to search for the target among distractors and report if the square was on the left or right side of the target by pressing the left or right arrow key on the keyboard. The search display remained on the screen for 5 s or until a response was made by the participants, whichever occurred earlier. If the participant made an error or did not respond, a short beep occurred immediately after the trial. After each trial, there was an inter-trial interval lasting 127 ms. Eye movements were not restricted or monitored. In the data analysis, we only included response time from the correct trials.

### Analyses

Model comparison was conducted by computing the AIC relative likelihood across the three models (Best Feature Guidance Model, Orthogonal Contrast Combination Model, Collinear Contrast Integration Model), using exp((AIC_min_ − AIC_i_)/2). Note that to make the AIC value of the Orthogonal Contrast Combination Model (winning model) comparable to the one from the Reciprocal of RT Model, we restricted the predictions to the same observations predicted by the Reciprocal of RT model, that is, the nine points observed at set size 20 across Experiments 2A–C.

## Supplementary Information

Supplementary Information.

## Data Availability

The data and code are available on OSF, link: https://osf.io/pkh68/.
