# Supplementary material for: Predicting how surface texture and shape combine in the human visual system to direct attention
Source: Sci Rep. 2021 Mar 17;11:6170. doi: 10.1038/s41598-021-85605-8 (PMC7971056; doi:10.1038/s41598-021-85605-8)
Supplement: Supplementary file 1 — Supplementary Information. [file 41598_2021_85605_MOESM1_ESM.docx]

Supplementary Information for

**Predicting how surface texture and shape combine in the human visual system to direct attention**

**Authors:** Zoe (Jing) Xu*^1^, Alejandro Lleras^1^, & Simona Buetti^1^

**Affiliations: ^1^** University of Illinois

***** **Corresponding author: Zoe (Jing) Xu**

Email: [jingxu9@illinois.edu](mailto:jingxu9@illinois.edu)

Phone: 1-217-979-8109

Address: 603 E. Daniel St., Champaign, IL 61820

**This PDF file includes:**

Supplementary Text

Figures S1

Table S1

References

**Supplementary Text**

**Limits of peripheral vision and stimuli selection considerations.**

Related to the constraints imposed by peripheral pooling^1-4^, in the current study we used surface textures that we believed peripheral vision would be able to differentiate. The results from the texture-search experiment (Experiment 1B) demonstrated that peripheral vision was indeed able to discriminate them. Figure S1 shows the RT by set size function for each target-distractor pairing when searching for texture (all shapes being equal). The functions were logarithmic, indicating that items were processed in parallel, with unlimited capacity ^5,6^. As a comparison, Figure S1 (left) shows the RT by set size function obtained in Experiment 1A for the shape-only search task. We can conclude that the type of surface texture we used can be processed in parallel by peripheral vision.


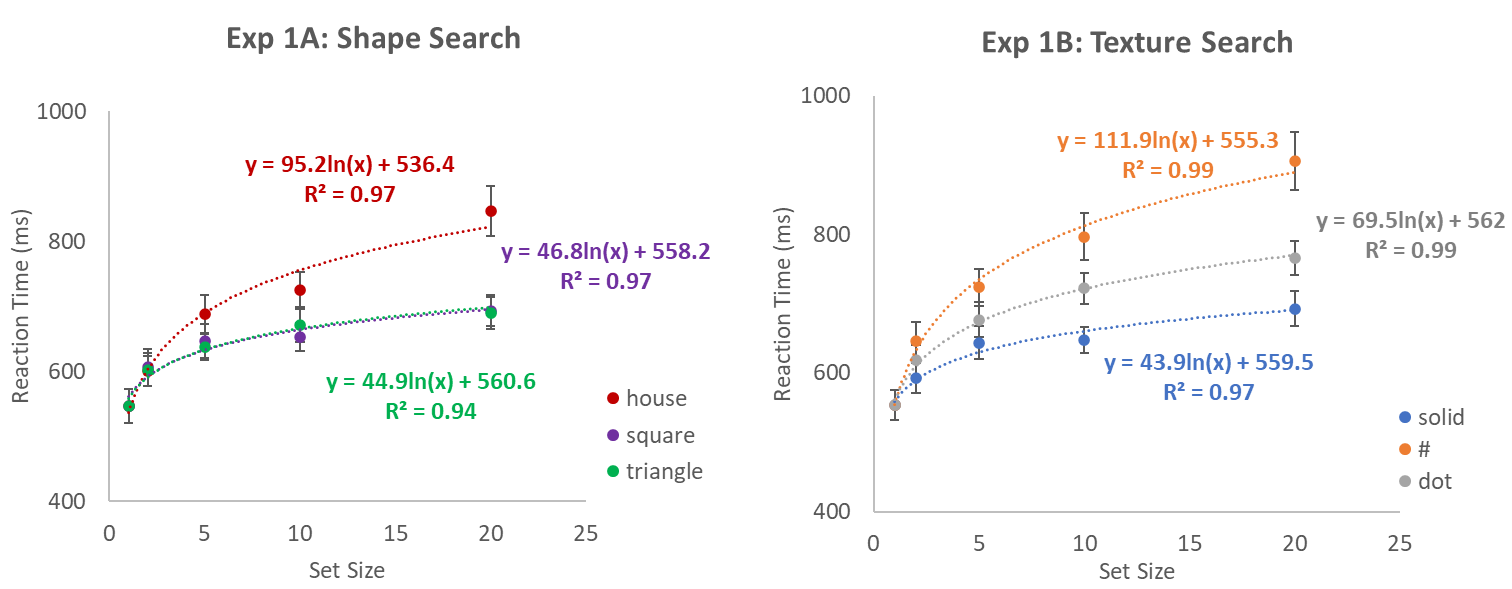


Figure S1. *Left*: Reaction times observed in the homogeneous search conditions of Experiment 1A as a function of distractor set sizes and distractor types when the target was an octagon and distractors were squares, triangles or houses. Results show that search efficiency, here indexed by the logarithmic search slope, varies as a function of target-distractor similarity. Search becomes less efficient when target-distractor similarity increases. *Right*: Reaction times observed in the homogeneous search conditions of Experiment 1B as a function of distractor set sizes and distractor types when the target was an octagon with white cross texture and distractors were octagons with tilted pound key, dotted or solid textures.

The question remains open regarding how more subtle material features (like the ones used in Cant et al.^7^ and Cavina-Pratesi et al.^8^) would be processed by peripheral vision. For instance, material features such as the texture of leather, bricks, and wood might not be distinguishable from one another in the periphery, once differences in overall color, luminance are equated for. If they are not, then it would be impossible to apply the approach used here simply because the information provided by these features would be insufficient to compute a contrast signal, and therefore, insufficient to guide attention. It is possible that certain material features can only be properly represented through foveal (or parafoveal) vision. If so, this might explain why studies using subtle material features might have found evidence for processing independence between shape and texture ^7,8^.

**Top-down contrast or bottom-up salience?**

In the attention literature, there are at least two ways to characterize the forces that guide attention in a scene: the bottom-up influence and the top-down influence. The bottom-up force is meant to reflect the extent to which local contrast surrounding an element in the scene *automatically* makes that element more salient to the observer and therefore more likely to attract attention, irrespective of the observer’s goals when viewing the scene^9-13^. The top-down influence is meant to reflect the extent to which information held in the observer’s mind guides attention to specific elements in the scene^14-17^. Target Contrast Signal Theory^18^ proposes that the top-down information that guides attention is the contrast computed by comparing the target template held in mind to the stimuli present in the scene. Sufficiently large contrast values allow the visual system to reject items in parallel that are unlikely to be the target. Visual attention is then directed to locations that were not rejected. Note that this account is similar to the relational theory^19-24^ that also proposes that attention is guided by a difference value and stands in direct contrasts to feature-centric theories of attention ^14,16,25^ that propose attention is guided toward the target by the relative match between features in the display and those of the target. According to these latter theories, when searching for a red item, the top-down guiding information is the color red. Items that match the specific color draw attention to themselves^16,17,26^. Those that do not are ignored by attention. Similar arguments are made in “optimal tuning” accounts of top-down guidance^25,27^.

The question that arises is: what is the source of attentional guidance in the experiments presented in this manuscript? Is it possible that attention is guided in a bottom-up fashion? Below we review evidence against this possibility. The traditional bottom-up contrast is computed as the visual difference between the features of an object and those of its immediate surroundings^10,11,13^. This computation can in theory occur over multiple spatial scales. Proponents of the bottom-up argument would say that when many distractors are present in the display, along with the target, the bottom-up contrast of the target is therefore impacted by the presence of those distractors. As distractor set size increases, it becomes more and more likely that distractors will appear near the target and therefore impact the computation of that contrast. In this view, at large set-sizes, the bottom-up contrast becomes the contrast between the target and the surrounding distractors. A consequence of this is that as set size increases, the additional cost to find the target diminishes because the additional distractors increase the target signal.

This proposal fails on several fronts. First, as discussed below, there are several reasons to believe that, at least at the stimulus densities studied here, the bottom-up contrast of the target is nothing more than the contrast of the target against its surrounding background. This is uncontroversial when the target is the only item in the display and this is also the case at very small set sizes, given that the likelihood of a distractor being close to the target is very small. If it is true that as set size increases, the target-distractor bottom-up signal increases in strength, then one would expect that the mechanisms that determine performance at large set sizes (when this contrast is maximal) would not be able to explain performance at the smaller set sizes (when this contrast is absent). More specifically, this account would predict that search efficiency should improve at larger set sizes, compared to smaller set sizes. In other words, the logarithmic slopes computed over large set size values should be significantly smaller than the logarithmic slopes computed over the small set sizes. This was first empirically tested in Buetti et al.^5^. The authors compared the slope of the logarithmic search function when it was computed based only on small set sizes to the slope computed based only on large set sizes. The two values of the slopes did not differ significantly from one another, suggesting that there was not an additional factor (like the hypothesized target-distractor bottom-up signal) that came into play at large set sizes. Instead, this analysis suggested that the same mechanisms governing behavior at small set sizes continue to govern behavior at large set sizes. As mentioned before, we propose that mechanism is the top-down contrast signal. We ran a similar analysis on all fifteen search functions reported in the present manuscript, comparing search slopes over small set sizes to search slopes over large set sizes. Note that here we only tested 4 distractor total sizes, instead of 5 used in Buetti et al.^5^ and that the largest set size in the present experiments was 20, as compared to 32. Therefore, the analysis from Buetti et al.^5^ allowed for a stronger test of this hypothesis given that each slope was fitted through three points at large set sizes (improving the fit of the estimated slope) and that larger set sizes were included, where the bottom-up salience signal ought to have been even stronger.

In the current analysis, to compute the slope over small set sizes, we fitted a logarithmic function through the small set sizes (set sizes 1-10). To compute the slope over large set sizes, we fitted a logarithmic function only over the two largest set sizes (10 and 20). Granted, fitting a logarithmic curve through two points (10 and 20) is less than optimal when trying to estimate the underlying search function. Nevertheless, for the purposes of this analysis, it seemed the most conservative thing to do: compute search efficiency computed exclusively over the large set sizes, where the target-distractor bottom-up signal should be maximally at play, and compare it to efficiency computed on the small set sizes where this factor is likely absent. As shown in Table S1 and consistent with the Buetti et al.^5^ analysis, there was no systematic evidence of increased search efficiency at large set sizes compared to small set sizes. Fourteen out of fifteen times, this was not the case: the slopes computed over large set sizes were not significantly smaller than the ones computed over small set sizes, except in Experiment 1A, when the distractor was a triangle shape. In sum, it is unlikely that different mechanisms are operating over small and large set sizes as there does not appear to be systematic evidence that the slopes at larger set sizes are smaller than the ones computed at lower set sizes.

Table S1. Logarithmic slope for the fifteen search functions computed over small set sizes (1-10) and over large set sizes (10-20) in Experiments 1-2. The asterisk indicates the only slope that was significantly smaller at larger set sizes compared to the smaller set sizes (at the alpha = 0.05 level).

| Experiment | distractor type | set size 1-10 | set size 10-20 |
| --- | --- | --- | --- |
| Experiment 1A | house | 80.1 | 175.3 |
|  | square | 46.0 | 58.9 |
|  | triangle | 52.1 | 27.0* |
| Experiment 1B | solid | 42.7 | 65.7 |
|  | tilted # | 102.7 | 157.0 |
|  | dots | 71.9 | 63.3 |
| Experiment 2A | house # | 63.0 | 85.9 |
|  | square dots | 28.7 | 38.2 |
|  | triangle solid | 37.5 | 11.2 |
| Experiment 2B | house dots | 47.8 | 63.3 |
|  | square solid | 21.2 | 18.2 |
|  | triangle # | 39.9 | 24.4 |
| Experiment 2C | house solid | 38.0 | 40.1 |
|  | square # | 49.6 | 35.2 |
|  | triangle dots | 40.6 | 42.8 |

Second, the idea that, if present, a bottom-up contrast would somehow facilitate responses is contradicted by extant data. In Buetti et al.^5^, we directly tested this hypothesis by comparing RTs in identical displays using two different search tasks: a “top-down” task, where the target defining features were known to the participants and fixed throughout the experiment, and a “bottom-up” task, where the target defining feature changed randomly from trial to trial. RTs in the “top-down” task were systematically faster than in the “bottom-up” task. The difference was particularly large at small set sizes, the largest difference being in the order of 150 ms at set size 3. Thus, performance in tasks that are presumably driven entirely by the bottom-up signal was in fact *slower* (not faster) than in tasks driven by the top-down contrast, at least at the display densities used in Buetti et al.^5^, which were actually denser than the displays used here. Tseng et al.^28^ proposed a model to account for the slow-down in the bottom-up task. Precisely because there is little-to-no target-distractor bottom-up contrast to drive attention, performance in bottom-up tasks is driven by two successive computations. An initial computation where the observer has to find which is the feature that is common to all distractors, and a second computation where the observer guides attention *away* from that color. The first computation is time consuming and is particularly difficult at small set sizes, but as set size increases, it becomes easier and easier to determine which feature is common to all distractors. In fact, the cost decreases as a function of 2/n, with n being set size. This results in an RT function that decreases as a power function of set size, which is fundamentally different than the monotonically increasing logarithmic functions observed when the target is known (top-down search).

Third, the proposal that the bottom-up target-distractor contrast determines performance in our search tasks would face difficulties accounting for certain patterns in the data. For instance, as we mentioned above, the bottom-up signal for the target is unlikely to be modulated by any featural information from the distractors at the smallest set sizes. This follows because at smaller set sizes, the distractors are overwhelmingly far from the target. Such an account would therefore predict that target-distractor similarity would only start modulating performance at the larger set sizes. This is contradicted by the data presented here, as well as in many previous papers^5,29-31^ where we show that even at the smallest set sizes, even when a single distractor is added to the display (set size 2), RTs are *already* modulated by target-distractor similarity. This sensitivity to target-distractor similarity at small set sizes is entirely consistent with the top-down account: each item has to be compared to the target template and will take different amounts of time to be rejected as a function of its level of similarity to the target. Thus, RTs at set size 2 will be much faster if the second item (the distractor) is very different from the target and will be relatively slower if the second item is very similar to the target. The pattern of data observed in distractor-heterogeneous displays is also difficult to explain in terms of bottom-up processes. Indeed, studies^29,32,33^ have shown that performance under distractor heterogeneous conditions can be entirely predicted in terms of the performance observed in distractor homogeneous conditions. The predictions for RTs observed in heterogeneous conditions are made using just two sets of parameters: the logarithmic slopes for each target-distractor pairing measured under homogeneous conditions and the number of each type of distractor present in the display. The success of these predictions, based on just these two sets of parameters, suggests that homogeneous and heterogeneous searches are governed by the same underlying mechanism: each item is independently compared to the target, in parallel, across the display, and is rejected at its own rate (which is set by its own level of similarity to the target). We believe an account based solely on bottom-up information would face difficulties explaining the success of these predictions.

In sum, there is little reason to believe that a bottom-up target-distractor salience signal drives attention in the experiments presented here. Rather, we propose it is the knowledge of the target features ahead of time that allows observers to efficiently process the display and orient to the target. We propose this is because the knowledge of the target features allow for the computation of a top-down contrast for each element in the display. This difference signal allows the visual system to discard, in parallel, distractors that are sufficiently different from the target.

**References**

1 Portilla, J. & Simoncelli, E. P. A parametric texture model based on joint statistics of complex wavelet coefficients. *International journal of computer vision* **40**, 49-70 (2000).

2 Freeman, J. & Simoncelli, E. P. Metamers of the ventral stream. *Nature neuroscience* **14**, 1195-1201 (2011).

3 Rosenholtz, R., Huang, J., Raj, A., Balas, B. J. & Ilie, L. A summary statistic representation in peripheral vision explains visual search. *Journal of vision* **12**, 14-14 (2012).

4 Rosenholtz, R. Capabilities and limitations of peripheral vision. *Annu Rev Vis Sci* **2**, 437-457, doi:10.1146/annurev-vision-082114-035733 (2016).

5 Buetti, S., Cronin, D. A., Madison, A. M., Wang, Z. & Lleras, A. Towards a better understanding of parallel visual processing in human vision: Evidence for exhaustive analysis of visual information. *J Exp Psychol Gen* **145**, 672-707, doi:10.1037/xge0000163 (2016).

6 Townsend, J. T. & Ashby, F. G. *Stochastic modeling of elementary psychological processes*. (CUP Archive, 1983).

7 Cant, J. S., Large, M.-E., McCall, L. & Goodale, M. A. Independent processing of form, colour, and texture in object perception. *Perception* **37**, 57-78 (2008).

8 Cavina-Pratesi, C., Kentridge, R., Heywood, C. & Milner, A. Separate channels for processing form, texture, and color: evidence from fMRI adaptation and visual object agnosia. *Cerebral cortex* **20**, 2319-2332 (2010).

9 Theeuwes, J. Perceptual selectivity for color and form. *Perception & psychophysics* **51**, 599-606 (1992).

10 Nothdurft, H.-C. Salience from feature contrast: additivity across dimensions. *Vision research* **40**, 1183-1201 (2000).

11 Itti, L. & Koch, C. Feature combination strategies for saliency-based visual attention systems. *Journal of Electronic imaging* **10**, 161-169 (2001).

12 Weidner, R. & Müller, H. J. Dimensional weighting in cross-dimensional singleton conjunction search. *Journal of Vision* **13**, 25-25 (2013).

13 Tudge, L., Brandt, S. A. & Schubert, T. Salience from multiple feature contrast: Evidence from saccade trajectories. *Attention, Perception, & Psychophysics* **80**, 677-690 (2018).

14 Bundesen, C. A theory of visual attention. *Psychological review* **97**, 523 (1990).

15 Folk, C. L., Remington, R. W. & Johnston, J. C. Involuntary covert orienting is contingent on attentional control settings. *Journal of Experimental Psychology: Human perception and performance* **18**, 1030 (1992).

16 Wolfe, J. M. Guided search 2.0 a revised model of visual search. *Psychonomic bulletin & review* **1**, 202-238 (1994).

17 Palmer, J., Ames, C. T. & Lindsey, D. T. Measuring the effect of attention on simple visual search. *Journal of Experimental Psychology: Human Perception and Performance* **19**, 108 (1993).

18 Lleras, A. *et al.* A target contrast signal theory of parallel processing in goal-directed search. *Atten Percept Psychophys* **82**, 394-425, doi:10.3758/s13414-019-01928-9 (2020).

19 Becker, S. I. Can intertrial effects of features and dimensions be explained by a single theory? *Journal of Experimental Psychology: Human Perception and Performance* **34**, 1417 (2008).

20 Becker, S. I. The role of target–distractor relationships in guiding attention and the eyes in visual search. *Journal of Experimental Psychology: General* **139**, 247 (2010).

21 Becker, S. I. Simply shapely: Relative, not absolute shapes are primed in pop-out search. *Attention, Perception, & Psychophysics* **75**, 845-861 (2013).

22 Becker, S. I., Folk, C. L. & Remington, R. W. Attentional capture does not depend on feature similarity, but on target-nontarget relations. *Psychological Science* **24**, 634-647 (2013).

23 Becker, S. I., Harris, A. M., Venini, D. & Retell, J. D. Visual search for color and shape: When is the gaze guided by feature relationships, when by feature values? *Journal of Experimental Psychology: Human Perception and Performance* **40**, 264 (2014).

24 Becker, S. I., Harris, A. M., York, A. & Choi, J. Conjunction search is relational: Behavioral and electrophysiological evidence. *Journal of Experimental Psychology: Human Perception and Performance* **43**, 1828 (2017).

25 Navalpakkam, V. & Itti, L. Search goal tunes visual features optimally. *Neuron* **53**, 605-617 (2007).

26 Hoffman, J. E. A two-stage model of visual search. *Perception & Psychophysics* **25**, 319-327 (1979).

27 Scolari, M. & Serences, J. T. Basing perceptual decisions on the most informative sensory neurons. *Journal of neurophysiology* **104**, 2266-2273 (2010).

28 Tseng, Y.-C., Glaser, J. I., Caddigan, E. & Lleras, A. Modeling the effect of selection history on pop-out visual search. *PLoS One* **9**, e89996 (2014).

29 Wang, Z., Buetti, S. & Lleras, A. Predicting search performance in heterogeneous visual search scenes with real-world objects. *Collabra: Psychology* **3** (2017).

30 Madison, A. M., Lleras, A. & Buetti, S. The role of crowding in parallel search: Peripheral pooling is not responsible for logarithmic efficiency in parallel search. *Attention, Perception, & Psychophysics* **80**, 352-373 (2018).

31 Wang, Z., Lleras, A. & Buetti, S. Parallel, exhaustive processing underlies logarithmic search functions: Visual search with cortical magnification. *Psychon Bull Rev* **25**, 1343-1350, doi:10.3758/s13423-018-1466-1 (2018).

32 Xu, J., Lleras, A. & Buetti, S. (revise and resubmit) Distractor-distractor interactions in visual search for oriented targets explain the increased difficulty observed in non-linearly separable conditions. *Journal of Experimental Psychology: Human Perception and Performances*.

33 Lleras, A., Wang, Z., Madison, A. M. & Buetti, S. Predicting search performance in heterogeneous scenes: Quantifying the impact of homogeneity effects in efficient search. *Collabra: Psychology* **5** (2019).
